# Significant changes in circulating microRNA by dietary supplementation of selenium and coenzyme Q10 in healthy elderly males. A subgroup analysis of a prospective randomized double-blind placebo-controlled trial among elderly Swedish citizens

**DOI:** 10.1371/journal.pone.0174880

**Published:** 2017-04-27

**Authors:** Urban Alehagen, Peter Johansson, Jan Aaseth, Jan Alexander, Dick Wågsäter

**Affiliations:** 1 Division of Cardiovascular Medicine, Department of Medical and Health Sciences, Linköping University, Linköping, Sweden; 2 Research Department, Innlandet Hospital Trust, Elverum, Norway, and Hedmark University College, Elverum, Norway; 3 Norwegian Institute of Public Health, Oslo, and Norwegian University of Life Sciences (NMBU), Ås, Norway; 4 Division of Drug Research, Department of Medical and Health Sciences, Faculty of Health Sciences, Linköping University, Linköping, Sweden; Indiana University Richard M Fairbanks School of Public Health, UNITED STATES

## Abstract

**Background:**

Selenium and coenzyme Q10 is essential for important cellular functions. A low selenium intake is reported from many European countries, and the endogenous coenzyme Q10 production is decreasing in the body with increasing age. Supplementation with selenium and coenzyme Q10 in elderly have shown reduced cardiovascular mortality and reduced levels of markers of inflammation. However, microRNA analyses could give important information on the mechanisms behind the clinical effects of supplementation.

**Methods:**

Out of the 443 healthy elderly participants that were given supplementation with 200 μg Se/day as organic selenium yeast tablets, and 200 mg/day of coenzyme Q10 capsules, or placebo for 4 years, 25 participants from each group were randomized and evaluated regarding levels of microRNA. Isolation of RNA from plasma samples and quantitative PCR analysis were performed. Volcano- and principal component analyses (PCA)–plots were used to illustrate the differences in microRNA expression between the intervention, and the placebo groups. Serum selenium concentrations were measured before intervention.

**Findings:**

On average 145 different microRNAs out of 172 were detected per sample. In the PCA plots two clusters could be identified indicating significant difference in microRNA expression between the two groups. The pre-treatment expression of the microRNAs did not differ between active treatment and the placebo groups. When comparing the post-treatment microRNAs in the active and the placebo groups, 70 microRNAs exhibited significant differences in expression, also after adjustment for multiple measurements. For the 20 microRNAs with the greatest difference in expression the difference was up to more than 4 fold and with a P-value that were less than 4.4e^-8^.

**Conclusions:**

Significant differences were found in expression of more than 100 different microRNAs with up to 4 fold differences as a result of the intervention of selenium and coenzyme Q10 combined. The changes in microRNA could be a part of mechanisms underlying the clinical effects earlier reported that reduced cardiovascular mortality, gave better cardiac function, and showed less signs of inflammation and oxdative stress following the intervention. However, more research is needed to understand biological mechanisms of the protective effects of selenium and Q10 supplementation.

## Introduction

Selenium (Se) is essential for a number of cellular functions. In the human selenoproteome 25 separate genes encodes for different selenoproteins[1]. Of those the intracellular glutathione peroxidase (GPX)-family including phospholipid hydroperoxide-, and gastrointestinal GPX, thioredoxin reductases (TXNRD) and extracellular selenoprotein P (SEPP 1) are important redox enzymes with a multitude of cellular and extracellular functions such as an anti-oxidative role [2, 3] as well as endothelial protection[4].

About half of the selenium in whole blood is found in plasma, of which GPX-3 constitutes 25% and SEPP1 usually constitutes more than 60% [5]. SEPP1 is a main transporter of selenium from the liver to peripheral tissues [6]. Hurst et al. demonstrated that an intake of about total 100 μg/d of selenium was needed in an adult UK population to achieve an optimal expression of SEPP1, thus a supplemental intake of 50 μg/d was needed, in addition to the habitual intake of ~55 μg/d in order to obtain an optimal expression of the selenoprotein [7]. However the intake of selenium differs in various parts of the world [4] mainly due to varying selenium content in the soil [4]. For example, a significantly higher content of selenium could be found in the soils in North America than in Europe [4] with reported serum selenium levels of US citizens generally above 120 μg/L[8, 9], whereas in several European countries including the Nordic countries (Sweden, Norway, and Denmark), levels below 90 μg/L have been reported. In Finland, the introduction of fertilizers with selenium have resulted in increased and presumably adequate levels[10]. Our group has recently reported mean serum selenium levels of 67.1 μg/L in a healthy elderly Swedish population, corresponding to a low selenium intake. This low intake was associated with an increased risk for cardiovascular mortality [11]. Apparently optimal level of selenium in the body is important for human health.

Besides multiple selenoproteins, coenzyme Q10 is also one of the important antioxidants in the body [12]. The functions of these two classes are interrelated; the selenoenzyme TrxR1 being needed for the cell to reduce coenzyme Q10 (ubiquinone) to its active form ubiquinol [13, 14]. Moreover, the synthesis of the selenocysteine-containing proteins requires a functional mevalonate pathway, of which coenzyme Q10 also is a product [15]. Because of a decline in the endogenous production of coenzyme Q10, only half of the production persists in the myocardium at the age of 80 years[16]. Based on these facts, an intervention with selenium and coenzyme Q10 was performed in healthy elderly community members for four years in a Swedish municipality. The results; a reduced cardiovascular mortality, improved cardiac function, and less increase of the biomarker NT-proBNP are previously reported [17]. Furthermore the monitoring of the biomarkers sP-selectin, hsCRP, copeptin, and MR-proADM in blood samples showed signs of less inflammatory activation [18], and less oxidative stress [19] respectively. Supplementation with selenium and coenzyme Q10 reduced the mortality particularly among those having the lowest selenium concentrations at baseline [20].

MicroRNAs, discovered in the beginning of 1990s [21, 22], provide a new field of research, and today more than 2000 different microRNAs are known within the human genome [23, 24]. However, their role in diseases have only partially been evaluated, and the literature on their associations to cardiac diseases is sparse. The microRNAs play key roles in the regulation of the mammalian protein-encoding genes, which includes genes important for disease processes, healing as well as the production of selenoproteins. If the effects of an intervention with selenium and coenzyme Q10 combined could be traced to have an impact on the regulation of microRNAs, this would increase the understanding about the underlying mechanisms for the protective effects of the intervention with selenium and coenzyme Q10 and act as a further validation of the clinical results from our main study referred to above[17].

The aim of the present study was to explore if the intervention with selenium and coenzyme Q10 influenced the expression of circulating microRNAs in the same population.

## Materials and methods

### Study population

The basal characteristics of the study population is presented in Table 1.

**Table 1:** Basal characteristics of the study population divided into active treatment and placebo.

	Active treatment	P-value	Placebo	Total study population
**n**	25		25	443
**Age years mean (SD)**	77.1 (3.5)	0.67	76.9 (3.1)	77.1 (3.5)
**History**				
**Smokers n (%)**	4 (16)	0.46	3 (12)	41 (9.3)
**Diabetes n (%)**	4 (16)	0.31	7 (28)	95 (21.4)
**Hypertension n (%)**	19 (76)	0.73	20 (80)	326 (73.6)
**IHD n (%)**	6 (24)	0.73	5 (20)	100 (22.6)
**NYHA class I n (%)**	16 (64)	0.39	13 (52)	226 (51.0)
**NYHA class II n (%)**	6 (24)	0.35	9 (36)	125 (28.2)
**NYHA class III n (%)**	6 (24)	0.27	3 (12)	88 (19.9)
**NYHA class IV n (%)**	0		0	0
**Unclassified NYHA n (%)**	0		0	4 (1.0)
**Medications**				
**ACEI n (%)**	3 (12)	0.27	6 (24)	89 (20.1)
**ARB n (%)**	0		4 (16)	23 (5.2)
**Betablockers n (%)**	8 (32)	0.15	13 (52)	153 (34.5)
**Digitalis n (%)**	0		0	22 (5.0)
**Diuretics n (%)**	8 (32)	0.56	10 (40)	158 (35.7)
**Statins n (%)**	5 (20)	1.0	5 (20)	96 (21.7)
**Examinations**				
**EF<40% n (%)**	3 (12)	0.64	2 (8)	33 (7.4)

Note: ARB; Angiotension receptor blockers; EF: Ejection fraction; IHD; Ischemic heart disease; NYHA: New York Heart Association functional class

Note: In the table the subpopulations evaluated through microRNA analyses divided into active treatment and placebo has been presented regarding basal characteristics and compared to the total study population for the clearness of representativity of the subpopulations.

In an epidemiological study in a rural municipality in Sweden that started in 1998, all participants in the age between 70–80 were invited to participate in the intervention study with selenium and coenzyme Q10. Of the 675 in this age span, 443 accepted participation in the study. All participants were examined by one of three experienced cardiologists. In all participants a new clinical history was recorded and a clinical examination was obtained. The New York Heart Association functional class (NYHA class) was also assessed, a Doppler-echocardiographical examination was carried out and an ECG was registered. Blood pressure was measured in the right arm with the participant resting in supine position. All participants were supplemented for 48 months, and were re-examined at the end of each six-month period. All-cause and cardiovascular mortalities were registered[17].

The study protocol has previously been published [25]. The Medical Product Agency declined reviewal of the study protocol since the study was not considered a trial of a medication for a certain disease but rather one of food supplement commodities that are commercially available. The study was approved by the Regional Ethical Committee (Diary no. 03–176) and conforms to the ethical guidelines of the 1975 Declaration of Helsinki. Written, informed consent was obtained from all patients.

The intervention study was registered at Clinicaltrials.gov, and has the identifier NCT01443780.

### Definitions

Diabetes mellitus was defined as having a fasting blood-glucose >7.0 mmol/L, or already undergoing treatment for diabetes (diet, oral therapy or insulin). Hypertension was defined as a resting blood pressure more than 140/90 mmHg as measured in the right arm or having a prior diagnosis of hypertension. Dyspnea was defined from the patient history, whereas presence of peripheral edema was defined from patient history and/or clinical examination [17]. Ischemic heart disease was defined as a history of angina pectoris and/or treatment for ischemic symptoms or on the basis of verified previous myocardial infarction. Cardiovascular mortality was defined as death caused by heart failure, and/or fatal arrhythmia, sudden death, ischemic heart disease or cerebrovascular disease.

### Intervention study

Between 2003 and 2010 a population consisting of 443 representative elderly individuals were randomized in blocks of 6 in a double-blind manner and given either the active intervention or placebo. Thus 221 persons were given supplement of selenium + coenzyme Q10 (200 μg Se/day given as organic selenium yeast tablets (SelenoPrecise 200 μg, Pharma Nord, Vejle, Denmark), and 200 mg/day of coenzyme Q10 capsules (Bio-Quinon 100 mg B.I.D, Pharma Nord, Vejle, Denmark), and 222 persons received the placebo supplement (placebo group) in addition to regular medication. The intervention time was 48 months, and the median follow-up period was 5.2 years.

### Study population for microRNA analysis

From the total study population of 443 participants, 25 male participants from the active treatment group, and 25 males from the placebo group were randomized and evaluated regarding microRNA, and constitutes the study population of the present study (Table 1). The microRNAs were analysed at the study start and at the study end in both the intervention and the placebo groups.

### Biochemical analyses

All blood samples were obtained while the patients were at rest in a supine position. The blood samples were collected in plastic vials containing EDTA (ethylenediamine tetracetic acid). The vials were placed on ice before chilled centrifugation at 3000g and then frozen at -70°C, and no sample was thawed more than twice.

### MicroRNA analysis

Isolation of RNA and real-time quantitative PCR analysis were performed by Exiqon A/S, Vedbaek, Denmark, as previously described [26].

### Sample preparation

Total RNA was extracted from serum using the miRCURY^™^ RNA isolation kit- biofluids (Exiqon A/S, Vedbaek, Denmark). In brief, 200μL of serum per sample, 60 μL of biofluids lysis solution containing 1μg carrier-RNA and RNA spike-in template mixture was added to the sample. 20μL protein precipitation solution was added and the tube was vortexed, incubated for 1 min at room temperature and centrifuged at 11,000g for 3 min. The clear supernatant was transferred to a new collection tube, and 270μL isopropanolol was added. The solutions were vortexed and transferred to a binding column. The column was incubated for 2 min at room temperature, and emptied using a vacuum-manifold. After that 100μL wash solution was added to the columns, the liquid was removed and 700μL wash solution was added. The liquid was removed using a vacuum-manifold. 250μL wash solution was added and the column was spun at 11,000g to dry the columns completely. The dry columns were transferred to a new collection tube and 50μL RNase free H_2_O was added directly on the membrane of the spin column to elute bound RNA. The column was incubated for 1 min at room temperature prior to centrifugation at 11,000g. The eluted RNA was stored in a -80°C freezer.

### MicroRNA real-time qPCR

Seven μL RNA was reverse transcribed in 35μL reactions using the miRCURY LNA^™^ UniversalRT microRNA PCR, Polyadenylation and cDNA synthesis kit (Exiqon A/S) [26]. cDNA was diluted 50x and assayed in 10μLPCR reactions according to the protocol for miRCURY LNA^™^ Universal RTmicroRNA PCR. Each microRNA was assayed once by qPCR on the microRNA Ready-to-use PCR, serum panel using ExiLENT SYBR^®^ Green master mix. The plasma panel includes 172 microRNAs validated to be significantly expressed in plasma. Negative controls excluding template from the reverse transcription reaction was performed and profiled like the samples. The amplification was performed in a LightCycler^®^ 480 Real-Time PCR System (Roche) in 384 well plates. The amplification curves were analysed using the Roche LC software, both for determination of Cq (by the 2^nd^ derivative method) and for the melting curve analysis.

### Data analysis

All assays were inspected for distinct melting curves and reactions with several melting points are removed from the data set. Reactions with amplification efficiency below 1.6, reactions with Cp values that are within 5 Cp of the negative controls and reactions with Cq>37 are removed from the dataset. microRNA-451 and 23a-3p were analysed to monitor hemolysis.Using NormFinder software, the best normalizer was found to be the average of assays detected in all samples. All data was normalized to the average of assays detected in all samples (average-assay Cq). 145 assays were detected on average per sample. Data analysis was performed according to Exiqon´s recommendation.

### Selenium analysis

The serum selenium analyses were performed using ICP-MS methodology at an Agilent 700 platform at Kompetenzzentrum für komplementärmedizinische Diagnostik, Zweigniederlassung der synlab MVZ Leinfelden GmbH (Leinfelden-Echterdingen, Germany). The accuracy of the measurements was checked by analyzing two external reference materials with certified values of 63 μg/L and 103 μg/L (control programme offered by the Society for Advancement of Quality Assurance in Medical Laboratories, INSTAND e.V., Düsseldorf, Germany), showing values within 90–110% of certified concentrations. A round-robin test with INSTAND e.V. always passed adequately. The precision of the method, checked by repetitive analyses of the same sera, showed an average coefficient of variation (CV) of 5.7%.

### Statistics

Descriptive data are presented as percentages or mean and the standard deviation (SD). P-values < 0.05 were considered significant, based on a two-sided evaluation.

A Principal Component Analysis (PCA) was used to reduce the dimension of the large data set obtained, and in a PCA plot, the 50 microRNAs that had the largest variation across all samples. The largest component in the variation is plotted along the X-axis, and the second largest is plotted on the Y-axis.

Data of microRNA are shown as fold changes between the groups. Two-group comparisons were performed using Student T-test and false discovery rate (FDR) adjustment using the Benjamini-Hochberg (BH) adjusted p-value, correcting for multiple testing. P-values < 0.05 were considered statistically significant.

A power calculation has been performed applying 25 participants in each group, and a type I error (alpha) of 0.05. Of those 101 mRNAs of the total 145 that obtained significant differences in mRNA expression, all had a power of >0,90, and more than 80% had a power of >0.95 in a two-tailed analysis.

All data were analyzed using standard software (Statistica v. 13, Statsoft Inc, Tulsa, OK, USA.).

## Results

### Baseline characteristics

The basal characteristics of the two groups, actively treated and placebo groups, for further evaluations of circulating microRNA are presented in Table 1. From this it could be seen that the two groups are well balanced. In the study population about 22% of the participants had a history of ischemic heart disease, about 77% had a diagnosed hypertension, and about 15% were smokers, which is about the same proportions as in the total study group consisting of 443 and therefore we regard the group evaluated with microRNAs as representative of an elderly “healthy” population in Sweden.

### MicroRNA evaluations

A PCR- panel was used for the study, and on average 145 microRNAs were detected per sample.

**Fig 1:**
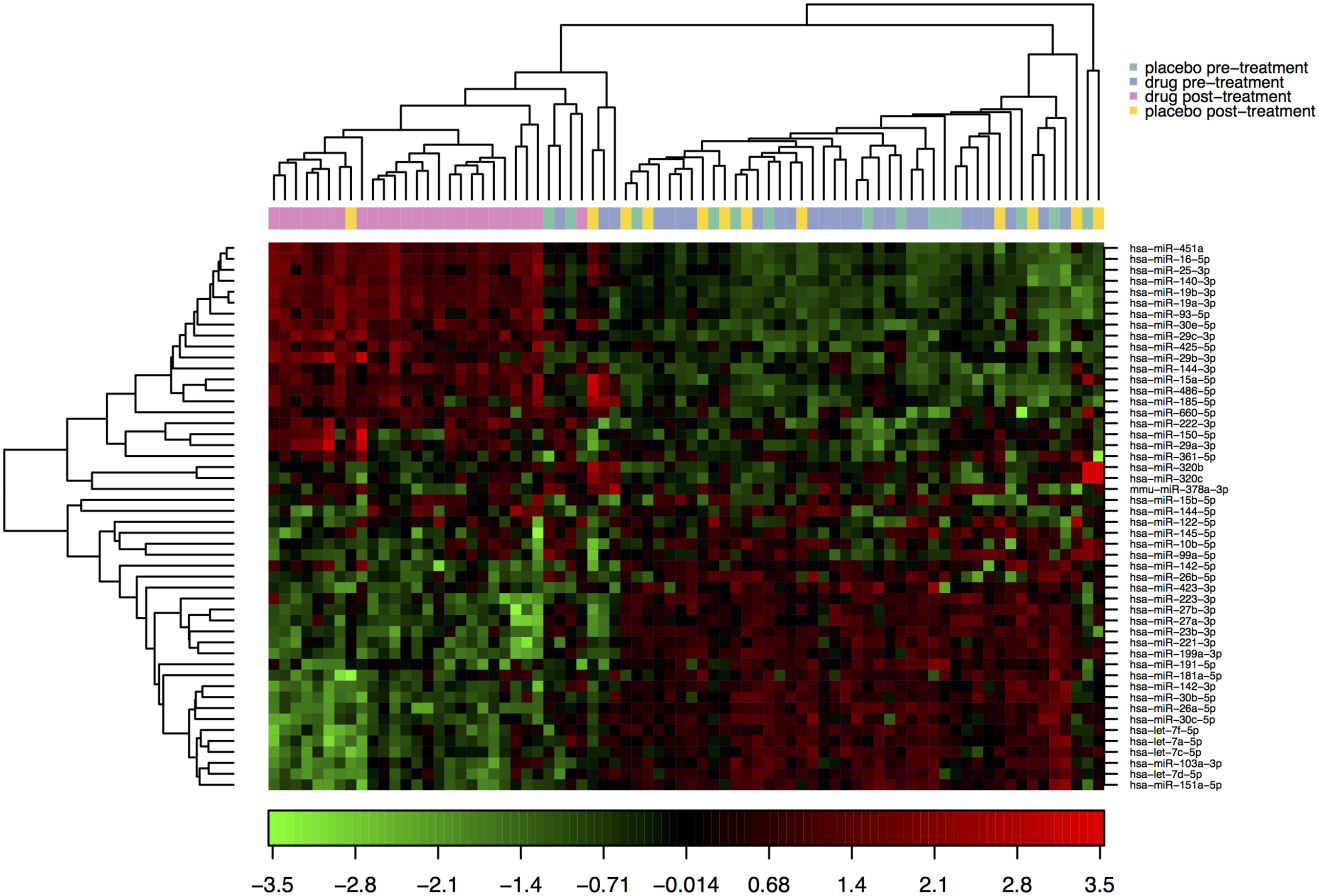
Heat map and hierarchical clustering of the study population in relation to micorRNA expression. Note: The clustering is performed on all samples, and on the top 50 microRNAs with the highest standard deviation. The normalized (dCq) values have been used for the analysis.

A heat map diagram was produced were the result of a two-way hierarchical clustering of the top 50 microRNAs with the highest standard deviation. The normalized values have been used and the samples are shown (Fig 1). In this, each row represents one microRNA, and each column represents one sample. The microRNA clustering could be seen on the left and includes the group receiving active intervention post treatment, whereas the others cluster in one group. The colour scale at the bottom illustrates the relative expression level of micro RNA across all samples, where red indicates an expression level above mean, and green represents a level lower than the mean. The microRNAs cluster in two groups; one being up- and the other being down-regulated with largely opposite expression in the post active-treatment group vs. the remaining groups.

### Principal Component Analysis plot

**Fig 2:**
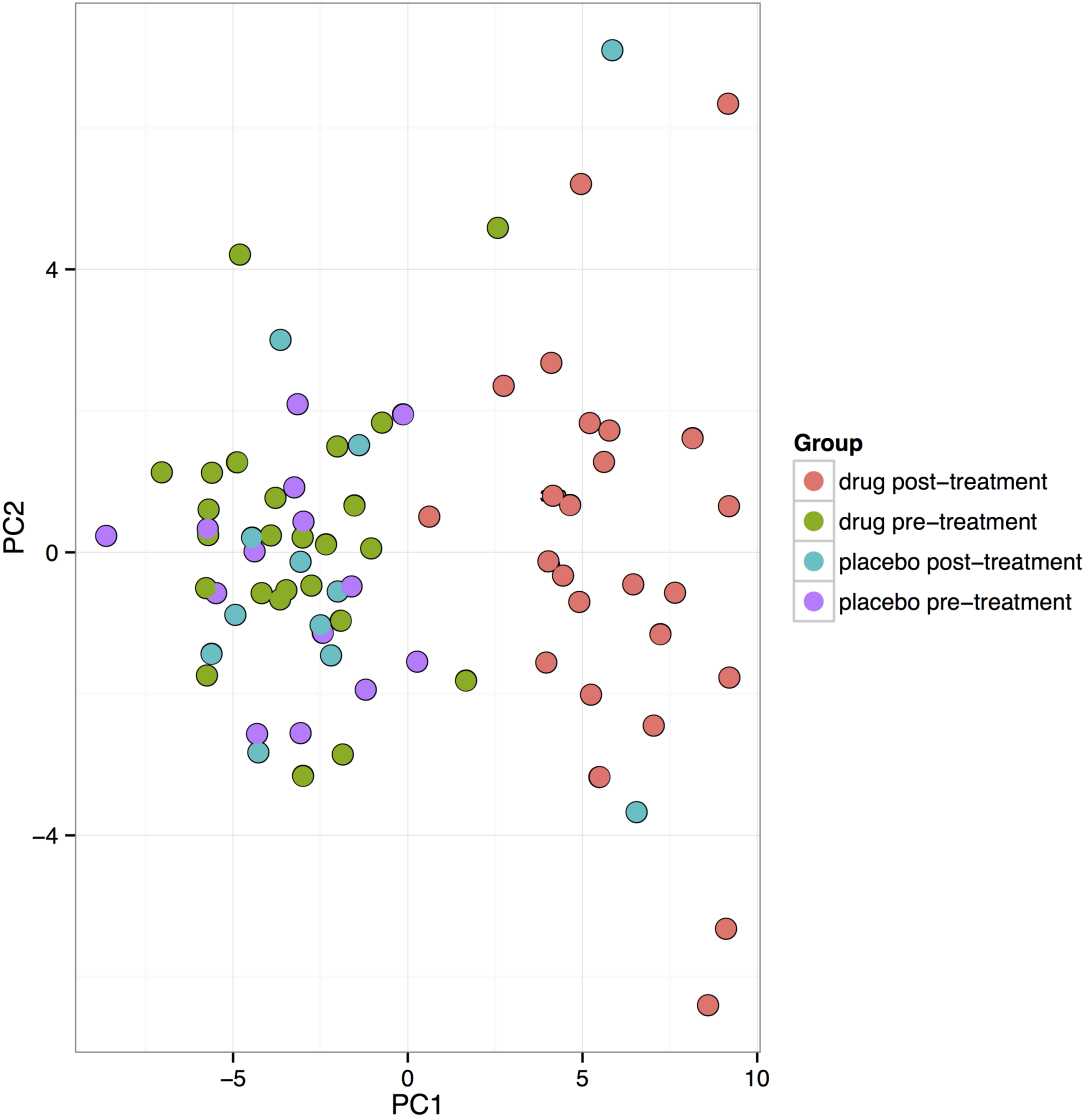
A Principal Component Analysis (PCA) plot of the study population illustrating the changes pre-treatment versus post-treatment in expression of microRNAs in those on intervention with selenium and coenzyme Q10 versus those on placebo. Note: The PCA analysis was performed on all samples, and on the top 50 microRNAs with the highest standard deviation. The normalized (dCq) values have been used for the analysis.

In the PCA plot (Fig 2), the 50 microRNAs that exhibited the largest variation across all samples were projected. The samples seems to cluster with little difference between the pre- and post-placebo treatment groups and pre-active treatment groups, whereas the post-active treatment samples cluster as a clearly separate group indicating that the intervention resulted in differences in microRNA expression.

### Differentially expressed microRNAs post-treatment active treatment group versus post-treatment placebo group

Comparing the post-treatment active group to the placebo group using Student´s *T*-test, 90 microRNAs were found to be differentially expressed using a cut-off *P*-value <0.05. Of those, 70 were still significant after correction for multiple measurements. S1 Table presents the *T*-values of all evaluated mRNAs.

In Table 2, the 20 microRNAs that were most differentially expressed are presented.

**Table 2:** Differential expressed microRNAs post-treatment active group versus post-treatment placebo group.

MicroRNA	Fold change	P-value	BH adj. P-value	Slope correlation coefficient
**Hsa-miR-199a-3p**	-2.6	1.1e^-7^	0.000016	-0.42
**Hsa-miR-26a-5p**	-2.9	1.8e^-7^	0.000016	-0.26
**Hsa-miR-199a-5p**	-5.0	9.3e^-7^	0.000055	-0.24
**Hsa-miR-221-3p**	-2.6	0.0000023	0.00010	-0.05
**Hsa-miR-151a-5p**	-2.0	0.0000041	0.00014	-0.32
**Hsa-miR-19b-3p**	3.5	0.0000071	0.00018	-0.40
**Hsa-miR-19a-3p**	3.3	0.0000072	0.00018	-0.17
**Hsa-miR-93-5p**	2.1	0.000013	0.00026	-0.20
**Hsa-miR-16-5p**	3.8	0.000013	0.00026	-0.28
**Hsa-miR-151a-3p**	-1.9	0.000039	0.00070	-0.42
**Hsa-miR-130a-3p**	-1.6	0.000045	0.00071	-0.51
**Hsa-miR-29b-3p**	2.2	0.000048	0.00071	-0.21
**Hsa-miR-30e-5p**	2.1	0.000053	0.00072	-0.17
**Hsa-miR-140-3p**	2.8	0.000058	0.00073	-0.03
**Hsa-miR-222-3p**	1.8	0.000061	0.00073	-0.25
**Hsa-miR-30c-5p**	-1.7	0.000078	0.00087	-0.07
**Hsa-miR-191-5p**	-1.5	0.00014	0.0015	-0.06
**Hsa-miR-363-3p**	2.7	0.00017	0.0016	-0.10
**Hsa-miR-125a-5p**	-2.7	0.00017	0.0016	-0.04
**Hsa-miR-451a**	3.4	0.00019	0.0017	-0.18

Note: BH adj: Benjamini-Hochberg adjusted *P*-value.

Note: The table shows the 20 microRNAs where the greatest difference could be demonstrated.

Note: The slope correlation coefficient indicates the relation between microRNA expression with regard to basal selenium concentration before intervention.

There were an increased expression of the microRNA as result of the intervention with selenium and coenzyme Q10 in the following microRNAs; miR-19b-3p, miR-93-5p, miR-16-5p, miR-29b-3p, miR-30e-5p, miR-140-3p, miR-22-3p, miR-363-3p and miR-451a, whereas the following microRNAs showed a decreased expression as a result of the intervention; miR-199a-3p, miR-26a-5p, miR-199a-5p, miR-221-3p, miR-151a-5p, miR-151a-3p, miR-130a-3p,miR-30c-5p, miR-191-5p, and miR-125a-5p.

As can be seen, all the 20 microRNAs have a highly significant difference in expression between the post treatment active treatment groups compared to the placebo group.

**Fig 3:**
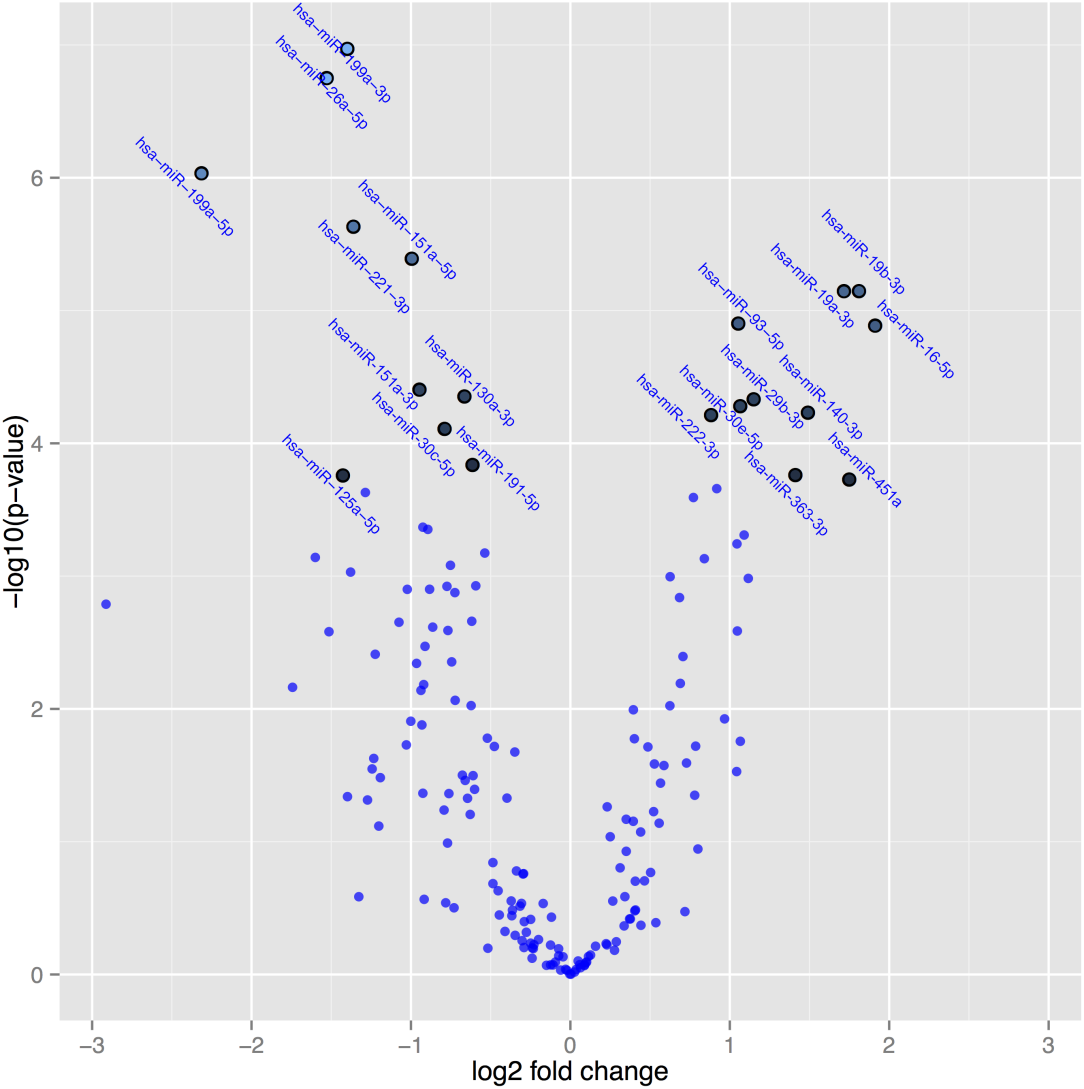
A volcano plot illustrating the relation between P-values of the changes in expression of microRNAs, and fold change in active treatment post-treatment versus placebo post-treatment. Note: The highlighted spots are microRNAs with P-values below 0.05 after Benjamini-Hochberg correction for multiple testing. The top 20 microRNAs are shown.

In Fig 3, a volcano plot, the differentially expressed microRNAs active post-treatment versus placebo post-treatment are presented. The volcano plot is constructed by plotting the *P*-values on the y-axis, and the fold change (ddCq) between the two experimental groups on the x-axis so that up- and down regulations appear equidistant from the centre. Plotting the values in this way results in two regions that show microRNAs with the highest magnitude of fold changes and high statistical significance. The highlighted spots are the 20 microRNAs with the greatest difference in expression and with a *P*-value<0.05 after correction for multiple measurements.

### Differentially expressed microRNAs pre-treatment active group versus pre-treatment placebo group

In order to evaluate that the above demonstrated changes partly could be explained by differences in microRNA expression already before intervention we have compared the pre-treatment active group to the pre-treatment placebo group using Student´s *T*-test. Only four microRNAs were found to be differentially expressed using a cut-off level of 0.05. However, after applying the Benjamini-Hochberger correction for multiple measurements, none of these differences persisted, indicating that no significant difference existed at the start of the intervention (Table 3).

**Table 3:** Differential expressed microRNAs post-treatment placebo group versus pre-treatment placebo group.

MicroRNA	Fold change	P-value	BH adj. P-value
**Hsa-miR-130a-3p**	1.4	0.0058	0.76
**Hsa-miR-194-5p**	2.3	0.011	0.76
**Hsa-miR-92b-3p**	-2.6	0.027	0.76
**Hsa-miR-27a-3p**	-1.4	0.041	0.76
**Hsa-miR-342-3p**	-1.5	0.0044	0.76
**Hsa-miR-382-5p**	-3.0	0.047	0.76

Note: BH adj: Benjamini-Hochberg adjusted *P*-value.

### Differentially expressed microRNAs post-treatment active treatment group versus pre-treatment active treatment group

Since the pre-treatment active group did not differ from the pre-treatment placebo group, as expected, we compared the microRNAs between the post-treatment and pre-treatment of the active group. From there 107 microRNAs were found to be differentially expressed. Applying correction for multiple measurements, still 101 microRNAs were differentially expressed after, compared with before treatment (Table 4). In general, the same microRNAs that demonstrated significant differences in expression between the intervention and the placebo groups could be found in Tables 2 and 4.

**Table 4:** Differential expressed microRNAs post-treatment active group versus pre-treatment placebo group.

MicroRNA	Fold change	P-value	BH-adj. P-value
**Hsa-miR-19b-3p**	3.7	3.3e^-19^	5.9e^-17^
**Hsa-miR-19a-3p**	3.5	1.2e^-17^	1.0e^-15^
**Hsa-miR-16-5p**	4.2	2.5e^-15^	1.5e^-13^
**Hsa-miR-451a**	3.8	8.3e^-13^	3.7e^-11^
**Hsa-miR-30e-5p**	2.8	2.7e^-12^	9.5e^-11^
**Hsa-miR-126-3p**	-1.9	6.4e^-12^	1.7e^-10^
**Hsa-miR-140-3p**	3.2	6.7e^-12^	1.7e^-10^
**Hsa-miR-29c-3p**	2.4	1.7e^-11^	3.7e^-10^
**Hsa-miR-151a-5p**	-2.4	2.4e^-11^	4.8e^-10^
**Hsa-miR-93-5p**	2.0	3.0e^-11^	5.1e^-11^
**Hsa-miR-26a-5p**	-3.7	3.2e^-11^	5.1e^-10^
**Hsa-miR-25-3p**	2.3	6.6e^-11^	9.8e^-10^
**Hsa-miR-22-3p**	1.8	1.6e^-10^	2.2e^-9^
**Hsa-miR-30b-5p**	-2.4	4.7e^-10^	5.9e^-9^
**Hsa-miR-16-2-3p**	2.9	5.0e^-10^	5.9e^-9^
**Hsa-miR-144-3p**	2.2	7.4e^-10^	8.2e^-9^
**Hsa-miR-486-5p**	2.2	2.7e^-10^	2.8e^-8^
**Hsa-let-7c-5p**	-2.8	3.2e^-9^	3.1e^-8^
**Hsa-miR-23b-3p**	-2.0	4.1e^-9^	3.9e^-8^
**Hsa-miR-199a-3p**	-2.5	5.0e^-9^	4.4e^-8^

Note: BH adj: Benjamini-Hochberg adjusted *P*-value.

Note: The table shows the 20 microRNAs where the greatest difference could be demonstrated.

**Fig 4:**
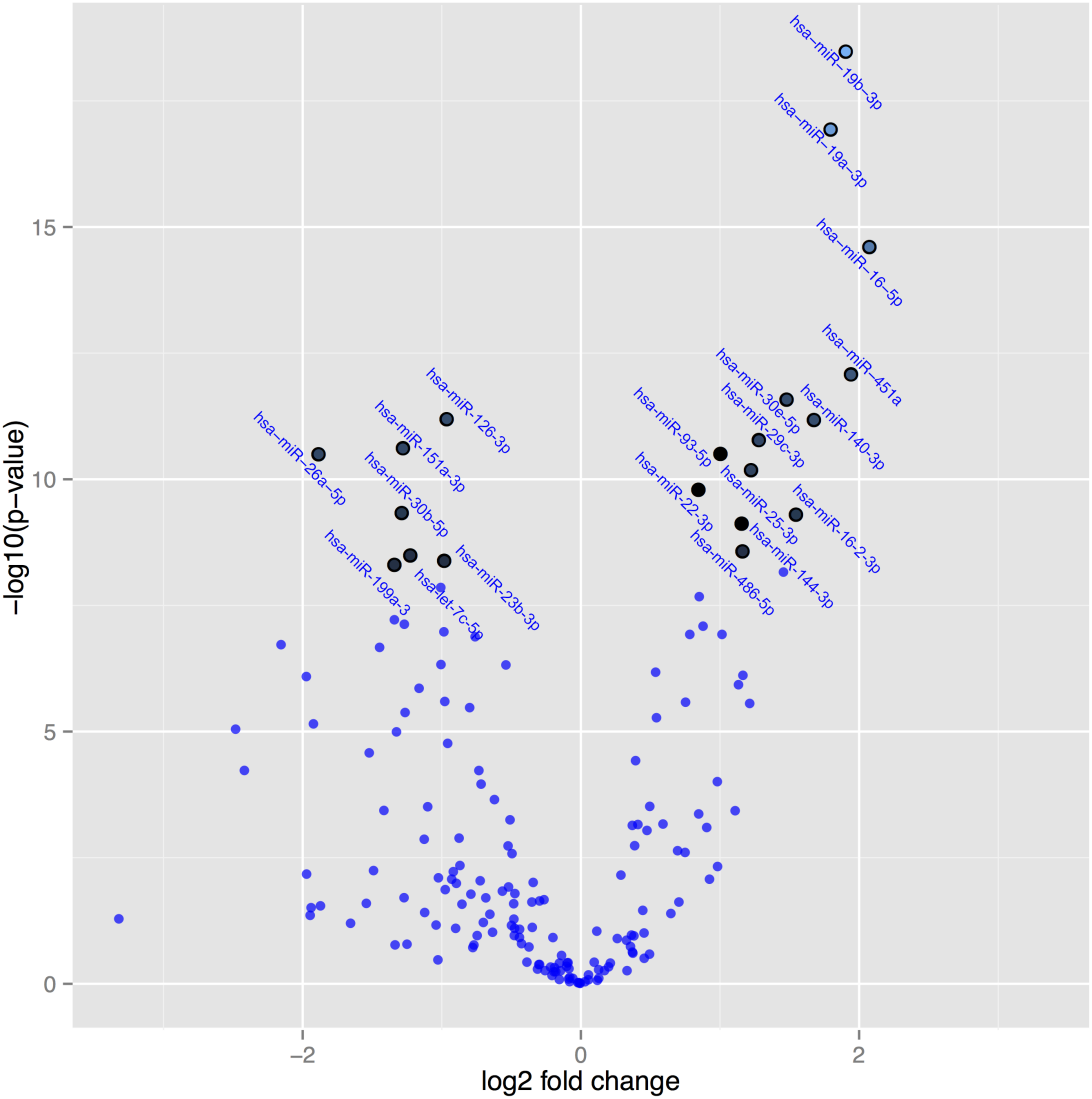
A volcano plot illustrating the relation between P-values of the changes in expression of microRNAs, and fold change in active treatment post-treatment versus active treatment pre-treatment. Note: The highlighted spots are microRNAs with P-values below 0.05 after Benjamini-Hochberg correction for multiple testing. The top 20 microRNAs are shown.

In Fig 4 the microRNAs are presented where significant difference in expression after correction for multiple measurements have been applied to the post-treatment and the pre-treatment active groups. The highlighted spots are the microRNAs with the greatest difference in expression and with a *P*-value <0.05 after correction for multiple measurements. As can be seen, highly significant differences in expression of several microRNAs can be demonstrated comparing the intervention with the placebo group (Fig 4).

### MicroRNA expression in relation to basal selenium concentration after intervention

In the study population where microRNA have been evaluated, the basal serum selenium levels were also measured. Serum selenium concentrations between 36.0 to 103.4 μg/L were observed, and the majority of participants had low selenium concentrations, as have recently been reported [11].

**Fig 5:**
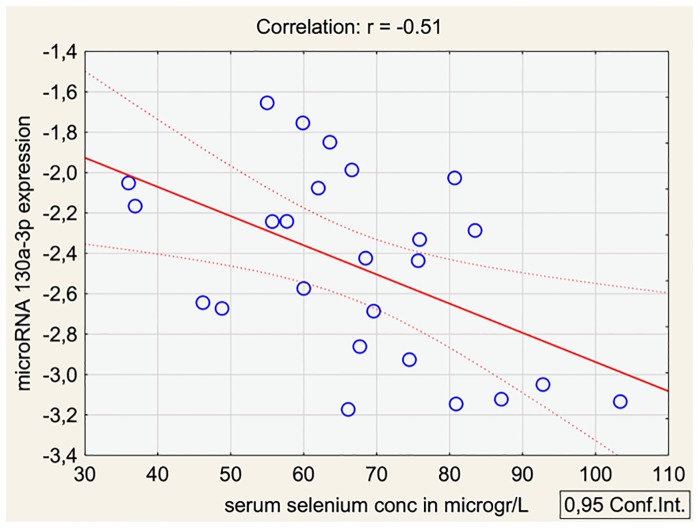
Scatterplot of expression of miRNA 130a-3p related to basal selenium concentration before intervention.

The microRNA expression differed depending on the basal selenium level in spite of the same intervention dosage of selenium, and where the correlation coefficient differed between -0.03 to -0.51. In all evaluated microRNAs the slope of the graph was negative, as illustrated in Fig 5.

## Discussion

### MicroRNA expression and intervention with selenium and coenzyme Q10

In the present study we examined whether intervention with selenium and coenzyme Q10 as supplements in elderly males during 4 years on top of their ordinary medication was associated with changes in circulating microRNA. We could present substantial effects in the expression of a multitude of microRNAs, and the observed changes remained even after adjustments for multiple measurements. We have previously reported that the dietary supplementation with selenium and coenzyme Q10 during four years led to reduced cardiovascular mortality, less age-dependent increase in the biomarker NT-proBNP [17], less oxidative stress[19], and less inflammatory activity[18]. In the present study 178 microRNAs (S1 Table) were detected per sample. However, we present only the 20 microRNAs where the most substantial differences could be seen when comparing the active with the placebo group. In the literature the most extensive information on microRNAs comes from evaluation of different types of tumour diseases. Changes in microRNA related to cardiac function or cardiovascular risk factors are sparse. However, some interesting findings are noted.

We observed a 2.2 fold increase in expression of microRNA-29b-3p as a result of the intervention, presumably reflecting a protective role of the extracellular matrix genes around the myocardium. Kriegel et al. presented data regarding the microRNA-29 family that has shown repressive effects on 16 *in vivo* confirmed extracellular matrix genes [27]. The different microRNA-29s are downregulated in the myocardium immediately after an infarction, as seen in mice. But during the healing process of the infarcted area, the expression of the extracellular matrix genes increases, and studies have shown a relationship between these genes and microRNA-29 [28].

A 2 fold increase of microRNA-30e-5p was found in our supplemented group compared with placebo. In a study on patients with acute heart failure, Ovchinnikova et al. found significantly decreased expression of microRNA-30e-5p, to about 50% of the levels found in controls [29], and Marfella et al. found an even more pronounced fall in expression of microRNA-30e-5p in heart failure patients compared with healthy controls [30]. Hence, increased expression could indicate raised protective protein production accompanied by diminished risk of heart failure, and presumably better cardiac function which accords with the clinical findings in the actively supplemented group previously reported [17].

Jickling et al. presented data showing that microRNA-19a had a decreased expression in patients with ischemic stroke [31]. In the present study a more than 3 fold increase in expression of the microRNA-19a-3p could be noted. This might also indicate a protective effect of the intervention.

We also found a decreased expression of several microRNAs that all appear to be involved also in cardiovascular disease processes. This accords with observations by e.g. Da Costa Martins et al. who found that increased expression microRNA-199a-5p was associated with myocardial hypertrophy [32]. Thus, a possible protective effect against myocardial hypertrophy by the intervention might be suggested.

Furthermore, we noticed a decrease in the expression of microRNA-130a-3p and microRNA-191-5p in the supplemented group to be compared with a 2.5 fold increase of microRNA-130a in patients with moderate pulmonary hypertension and a more than 9 fold increase in patients with severe pulmonary hypertension as reported by Wei et al. [33].

The changed expressions of microRNAs resulting from the intervention with selenium and coenzyme Q10 in the present study may provide mechanisms for cardioprotective effects. However, the different microRNAs presented are also expressed in many other disease states, for example microRNA-140-3p is less expressed in lung cancer patients compared to healthy controls[34]. And according to Yang et al. an inhibitory effect on breast cancer progression and metastasis of raised microRNA-19a-3p may result [35].

Thus, the changed expression of the different microRNAs observed after supplementation with selenium and coenzyme Q10 may protect against cardiovascular disease as well as cancer. However, as the research area is new and presently not completely covered, more data will be needed to fully elucidate the relation between intervention of selenium and coenzyme Q10 and specific microRNAs.

### MicroRNA expression and serum selenium concentration

We have recently reported that in 98% of the population evaluated in the present study a selenium deficiency defined as a serum concentration below the concentration for optimum function of the SEPP1 could be seen[20]. In an interesting publication Xing et al, reported upregulated microRNAs (miR-16, miR-199a-5p and miR-30e), and downregulated microRNAs (miR-450a, miR-675, and miR3571) from those with selenium deficiency in a rat model [36]. However, following a selenium supplementation a downregulation of microRNAs could be seen in the selenium deficient animals (miR-450a, miR-675 and miR3571, miR-675, miR-199a-5p, miR-16, miR195 and miR-30e), which is in agreement with the changes demonstrated in the majority of the microRNAs in the present human intervention study. It is so far demonstrated that the supplementation have clinical effects in terms of reduced inflammatory activity, less oxidative stress, and reduced cardiovascular mortality in comparison with placebo. Some of the mechanisms behind this could be found in the changes in expressions of microRNAs, which are responsible for gene regulation and ultimately production of proteins such as TXNRD or SEPP1. Our results thereby show that the effects of selenium and Q10 may be mediated at the molecular level through changes in microRNA expression.

We have also evaluated the basal selenium concentration to the fold change of the expression of the 20 microRNAs with the greatest fold change as a result of the intervention. To the knowledge of the authors, no such evaluation has previously been reported in the literature. It is interesting to note that even if the evaluated sample is small, the message is the same in a majority of the microRNAs analysed; a negative slope of the graph illustrating the fold change as the selenium concentration increases. This was both noted in the 10 microRNAs with increased expression as a result of the intervention, and in the 10 microRNAs with decreased expression (Table 2). The interpretation of this could be that the intervention was not enough to neutralize the deficiency in those with low basal selenium concentration, a fact that could be seen in those with no or low deficiency on selenium when given intervention. The result would therefore be that the microRNA expression differs between those low on selenium and those high on selenium before intervention, as could be seen in Fig 5.

## Limitations

The present report is based on male participants only, and with a restricted age span. Therefore the results could not automatically be extrapolated into other age classes, or even into female patients in the corresponding age span. However, we regard the data so interesting that the report should stimulate further research into other study groups.

Also, the study population consisted of a homogenous group of Caucasian males, a fact that also could restrict the extrapolation into other study groups.

A panel comprising the 172 microRNAs most commonly found in plasma was analysed. Out of the 2000 microRNA known to be expressed at higher levels in cells and tissues and less in plasma, we might have missed some.

Finally, the sample size of this study is small, especially the analysis of the microRNA expression depending on basal selenium concentration, however, it did not preclude finding some consistent changes in microRNA expression because of their magnitude.

Therefore we argue that the present report give new and important information.

## Conclusions

MicroRNAs are important factors in the regulation of protein-encoding genes. As demonstrated previously, there is a selenium deficiency in the Swedish population. The present study is an evaluation of the changes in expression of microRNAs as a result of intervention with selenium and coenzyme Q10 in a healthy elderly population. We could demonstrate resulting significant differences in expression of more than 100 different microRNAs with up to 4 fold differences. The changes observed are compatible with previous studies on changes in microRNA and cardiovascular diseases and selenium deficiency. These changes in microRNA could be a part of mechanisms underlying the clinical effects earlier reported for the same cohort, reduced cardiovascular mortality, better cardiac function, and less signs of inflammation and oxdative stress following the intervention. However, more research is needed to understand biological mechanisms of the protective effects of selenium and Q10 supplementation.

## Supporting information

S1 Table Differential expressed microRNAs post-treatment active group versus post-treatment placebo group, all microRNAs. (DOCX)
