# Supplementary material for: Significant changes in circulating microRNA by dietary supplementation of selenium and coenzyme Q10 in healthy elderly males. A subgroup analysis of a prospective randomized double-blind placebo-controlled trial among elderly Swedish citizens
Source: PLoS One. 2017 Apr 27;12(4):e0174880. doi: 10.1371/journal.pone.0174880 (PMC5407645; doi:10.1371/journal.pone.0174880)
Supplement: S1 Table — (DOCX) [file pone.0174880.s001.docx]

**Supplemental Table 1. Differential expressed microRNAs post-treatment active group versus post-treatment placebo group, all microRNAs**

| MicroRNA | SD drug post-treatment | SD placebo post-treatment | Average dcq drug post-treatment | Average dcq placebo post-treatment | ddcq | Fold change | t-test p-value | BH adj p-value |
| --- | --- | --- | --- | --- | --- | --- | --- | --- |
| 199a-3p | 0.53 | 0.51 | -2.86 | -1.46 | -1.40 | -2.64 | 1.1E-07 | 1.6E-05 |
| 26a-5p | 0.76 | 0.57 | -2.55 | -1.02 | -1.53 | -2.88 | 1.8E-07 | 1.6E-05 |
| 199a-5p | 1.43 | 0.74 | -4.88 | -2.57 | -2.31 | -4.97 | 9.3E-07 | 5.5E-05 |
| 221-3p | 0.93 | 0.53 | -1.48 | -0.12 | -1.36 | -2.57 | 2.3E-06 | 1.0E-04 |
| 151a-5p | 0.54 | 0.46 | -2.06 | -1.07 | -0.99 | -1.99 | 4.1E-06 | 1.4E-04 |
| 19b-3p | 0.42 | 0.85 | 4.07 | 2.26 | 1.81 | 3.51 | 7.1E-06 | 1.8E-04 |
| 19a-3p | 0.38 | 0.80 | 3.01 | 1.29 | 1.72 | 3.29 | 7.2E-06 | 1.8E-04 |
| 93-5p | 0.45 | 0.54 | 1.53 | 0.47 | 1.05 | 2.08 | 1.3E-05 | 2.6E-04 |
| 16-5p | 0.48 | 0.95 | 7.57 | 5.66 | 1.91 | 3.76 | 1.3E-05 | 2.6E-04 |
| 151a-3p | 0.74 | 0.46 | -4.10 | -3.15 | -0.95 | -1.93 | 3.9E-05 | 7.0E-04 |
| 130a-3p | 0.47 | 0.35 | -2.46 | -1.80 | -0.66 | -1.58 | 4.5E-05 | 7.1E-04 |
| 29b-3p | 0.79 | 0.61 | -1.77 | -2.91 | 1.14 | 2.20 | 4.8E-05 | 7.1E-04 |
| 30e-5p | 0.40 | 0.60 | 0.19 | -0.86 | 1.05 | 2.07 | 5.3E-05 | 7.2E-04 |
| 140-3p | 0.42 | 0.85 | 0.93 | -0.55 | 1.48 | 2.78 | 5.8E-05 | 7.3E-04 |
| 222-3p | 0.47 | 0.51 | 0.38 | -0.50 | 0.88 | 1.84 | 6.1E-05 | 7.3E-04 |
| 30c-5p | 0.48 | 0.46 | -1.80 | -1.01 | -0.79 | -1.73 | 7.8E-05 | 8.7E-04 |
| 191-5p | 0.61 | 0.27 | -1.42 | -0.81 | -0.61 | -1.53 | 1.4E-04 | 1.5E-03 |
| 363-3p | 0.47 | 0.91 | -0.67 | -2.08 | 1.41 | 2.66 | 1.7E-04 | 1.6E-03 |
| 125a-5p | 0.78 | 0.91 | -3.80 | -2.37 | -1.43 | -2.69 | 1.7E-04 | 1.6E-03 |
| 451a | 0.45 | 1.13 | 8.52 | 6.77 | 1.75 | 3.37 | 1.9E-04 | 1.7E-03 |
| 29c-3p | 0.43 | 0.61 | 0.23 | -0.68 | 0.92 | 1.89 | 2.2E-04 | 1.9E-03 |
| let-7d-5p | 0.95 | 0.81 | -3.12 | -1.83 | -1.29 | -2.44 | 2.3E-04 | 1.9E-03 |
| 20a-5p | 0.53 | 0.50 | 1.86 | 1.09 | 0.77 | 1.71 | 2.6E-04 | 2.0E-03 |
| 27b-3p | 0.56 | 0.65 | -0.74 | 0.18 | -0.93 | -1.90 | 4.3E-04 | 3.2E-03 |
| 223-3p | 0.84 | 0.54 | 3.06 | 3.95 | -0.89 | -1.86 | 4.4E-04 | 3.2E-03 |
| 25-3p | 0.54 | 0.78 | 1.66 | 0.57 | 1.09 | 2.13 | 4.9E-04 | 3.4E-03 |
| 144-3p | 0.43 | 0.77 | 3.43 | 2.39 | 1.04 | 2.06 | 5.7E-04 | 3.8E-03 |
| 652-3p | 0.39 | 0.39 | -2.34 | -1.81 | -0.54 | -1.45 | 6.7E-04 | 4.3E-03 |
| 766-3p | 1.22 | 0.88 | -6.12 | -4.52 | -1.60 | -3.03 | 7.2E-04 | 4.4E-03 |
| 425-5p | 0.44 | 0.63 | -0.18 | -1.02 | 0.84 | 1.79 | 7.4E-04 | 4.4E-03 |
| 126-3p | 0.44 | 0.57 | 1.59 | 2.34 | -0.75 | -1.68 | 8.3E-04 | 4.8E-03 |
| 301a-3p | 1.49 | 0.79 | -5.06 | -3.68 | -1.38 | -2.60 | 9.3E-04 | 5.2E-03 |
| 101-3p | 0.38 | 0.48 | 1.95 | 1.33 | 0.63 | 1.54 | 1.0E-03 | 5.4E-03 |
| 16-2-3p | 0.54 | 0.88 | -1.44 | -2.56 | 1.12 | 2.17 | 1.0E-03 | 5.4E-03 |
| 126-5p | 0.49 | 0.44 | -1.79 | -1.20 | -0.59 | -1.51 | 1.2E-03 | 5.9E-03 |
| 27a-3p | 0.68 | 0.57 | -1.60 | -0.82 | -0.77 | -1.71 | 1.2E-03 | 5.9E-03 |
| 30b-5p | 0.57 | 0.70 | -1.47 | -0.59 | -0.88 | -1.84 | 1.3E-03 | 5.9E-03 |
| 421 | 0.78 | 0.49 | -6.54 | -5.52 | -1.02 | -2.03 | 1.3E-03 | 5.9E-03 |
| let-7a-5p | 0.67 | 0.53 | 0.68 | 1.40 | -0.72 | -1.65 | 1.3E-03 | 6.1E-03 |
| 32-5p | 0.42 | 0.56 | -0.62 | -1.31 | 0.68 | 1.61 | 1.5E-03 | 6.5E-03 |
| 335-3p | 1.32 | 0.84 | -7.41 | -4.50 | -2.91 | -7.53 | 1.6E-03 | 7.1E-03 |
| 103a-3p | 0.53 | 0.50 | 0.50 | 1.12 | -0.62 | -1.54 | 2.2E-03 | 9.2E-03 |
| let-7e-5p | 0.98 | 0.86 | -4.47 | -3.39 | -1.08 | -2.11 | 2.2E-03 | 9.2E-03 |
| 142-3p | 0.70 | 0.72 | 1.59 | 2.45 | -0.86 | -1.82 | 2.4E-03 | 9.8E-03 |
| 145-5p | 0.82 | 0.57 | -3.00 | -2.24 | -0.77 | -1.70 | 2.6E-03 | 9.9E-03 |
| 15b-3p | 0.86 | 0.88 | -2.50 | -3.54 | 1.05 | 2.07 | 2.6E-03 | 9.9E-03 |
| 374b-5p | 1.21 | 1.26 | -5.55 | -4.04 | -1.51 | -2.86 | 2.6E-03 | 9.9E-03 |
| 331-3p | 0.90 | 0.74 | -4.33 | -3.42 | -0.91 | -1.88 | 3.4E-03 | 1.3E-02 |
| 326 | 1.20 | 0.92 | -5.29 | -4.06 | -1.22 | -2.34 | 3.9E-03 | 1.4E-02 |
| 192-5p | 0.38 | 0.67 | -1.71 | -2.42 | 0.71 | 1.63 | 4.0E-03 | 1.4E-02 |
| 23b-3p | 0.44 | 0.71 | -0.56 | 0.18 | -0.74 | -1.68 | 4.4E-03 | 1.5E-02 |
| let-7f-5p | 0.90 | 0.86 | -1.67 | -0.71 | -0.96 | -1.95 | 4.5E-03 | 1.6E-02 |
| 140-5p | 0.73 | 0.62 | -3.05 | -3.74 | 0.69 | 1.61 | 6.4E-03 | 2.2E-02 |
| let-7c-5p | 0.68 | 0.92 | -3.41 | -2.49 | -0.92 | -1.89 | 6.6E-03 | 2.2E-02 |
| 376c-3p | 1.18 | 1.64 | -6.33 | -4.58 | -1.74 | -3.35 | 6.9E-03 | 2.2E-02 |
| 335-5p | 1.12 | 0.58 | -7.49 | -6.56 | -0.94 | -1.91 | 7.3E-03 | 2.3E-02 |
| 18a-5p | 0.99 | 0.58 | -3.22 | -2.50 | -0.72 | -1.65 | 8.6E-03 | 2.7E-02 |
| 423-3p | 0.52 | 0.65 | -2.35 | -1.73 | -0.62 | -1.54 | 9.4E-03 | 2.9E-02 |
| 215-5p | 0.52 | 0.65 | -3.19 | -3.81 | 0.62 | 1.54 | 9.5E-03 | 2.9E-02 |
| 21-5p | 0.45 | 0.38 | 3.91 | 3.52 | 0.40 | 1.32 | 1.0E-02 | 3.0E-02 |
| 486-5p | 0.51 | 1.10 | 2.91 | 1.94 | 0.97 | 1.96 | 1.2E-02 | 3.5E-02 |
| 33a-5p | 1.35 | 0.91 | -3.97 | -2.97 | -1.00 | -2.00 | 1.2E-02 | 3.6E-02 |
| 28-5p | 0.76 | 0.89 | -5.09 | -4.16 | -0.93 | -1.91 | 1.3E-02 | 3.7E-02 |
| 26b-5p | 0.43 | 0.61 | -2.51 | -1.99 | -0.52 | -1.43 | 1.7E-02 | 4.6E-02 |
| 106a-5p | 0.52 | 0.41 | 1.12 | 0.72 | 0.40 | 1.32 | 1.7E-02 | 4.6E-02 |
| 2110 | 1.07 | 1.00 | -5.51 | -6.58 | 1.07 | 2.09 | 1.8E-02 | 4.7E-02 |
| 139-5p | 1.17 | 1.14 | -4.23 | -3.20 | -1.03 | -2.04 | 1.9E-02 | 4.9E-02 |
| 29a-3p | 0.96 | 0.86 | -0.12 | -0.90 | 0.79 | 1.72 | 1.9E-02 | 4.9E-02 |
| 23a-3p | 0.39 | 0.57 | 2.01 | 2.49 | -0.48 | -1.39 | 1.9E-02 | 4.9E-02 |
| 660-5p | 0.49 | 0.57 | -0.80 | -1.29 | 0.49 | 1.40 | 1.9E-02 | 4.9E-02 |
| 107 | 0.48 | 0.37 | -0.86 | -0.51 | -0.35 | -1.27 | 2.1E-02 | 5.3E-02 |
| 1 | 0.93 | 1.26 | -6.62 | -5.39 | -1.23 | -2.35 | 2.4E-02 | 5.8E-02 |
| 7-5p | 0.84 | 0.80 | -5.10 | -5.83 | 0.73 | 1.66 | 2.6E-02 | 6.2E-02 |
| 22-3p | 0.30 | 0.70 | 0.97 | 0.45 | 0.53 | 1.44 | 2.6E-02 | 6.3E-02 |
| 15a-5p | 0.35 | 0.78 | 4.11 | 3.53 | 0.59 | 1.50 | 2.7E-02 | 6.3E-02 |
| 485-3p | 0.33 | 1.01 | -6.23 | -4.99 | -1.24 | -2.37 | 2.8E-02 | 6.6E-02 |
| 532-3p | 0.71 | 1.25 | -3.77 | -4.81 | 1.04 | 2.06 | 3.0E-02 | 6.9E-02 |
| 125b-5p | 0.74 | 0.87 | -3.00 | -2.33 | -0.68 | -1.60 | 3.2E-02 | 7.2E-02 |
| 99a-5p | 0.59 | 0.80 | -2.79 | -2.18 | -0.61 | -1.53 | 3.2E-02 | 7.2E-02 |
| 495-3p | 1.00 | 1.23 | -6.48 | -5.29 | -1.19 | -2.28 | 3.3E-02 | 7.3E-02 |
| 152-3p | 0.93 | 0.79 | -3.72 | -3.06 | -0.66 | -1.58 | 3.5E-02 | 7.6E-02 |
| 590-5p | 0.54 | 0.77 | -2.11 | -2.68 | 0.57 | 1.48 | 3.6E-02 | 7.9E-02 |
| 100-5p | 1.00 | 0.68 | -4.33 | -3.73 | -0.60 | -1.52 | 4.0E-02 | 8.7E-02 |
| 338-3p | 1.25 | 1.18 | -4.88 | -3.95 | -0.93 | -1.90 | 4.3E-02 | 9.1E-02 |
| 374a-5p | 0.81 | 1.05 | -5.73 | -4.96 | -0.76 | -1.70 | 4.3E-02 | 9.1E-02 |
| 210-3p | 0.62 | 1.02 | -2.54 | -3.32 | 0.78 | 1.72 | 4.5E-02 | 9.3E-02 |
| 409-3p | 1.86 | 1.65 | -6.75 | -5.35 | -1.40 | -2.64 | 4.6E-02 | 9.4E-02 |
| let-7d-3p | 0.49 | 0.55 | -1.27 | -0.87 | -0.40 | -1.32 | 4.7E-02 | 9.4E-02 |
| 197-3p | 0.87 | 0.85 | -3.34 | -2.69 | -0.65 | -1.56 | 4.7E-02 | 9.4E-02 |
| 376a-3p | 1.21 | 1.68 | -6.37 | -5.10 | -1.27 | -2.42 | 4.9E-02 | 9.6E-02 |
| 106b-5p | 0.38 | 0.30 | -0.23 | -0.46 | 0.23 | 1.17 | 5.5E-02 | 1.1E-01 |
| 143-3p | 1.18 | 1.11 | -2.57 | -1.78 | -0.79 | -1.73 | 5.8E-02 | 1.1E-01 |
| 324-3p | 0.62 | 0.79 | -2.67 | -3.19 | 0.52 | 1.44 | 5.9E-02 | 1.1E-01 |
| 328-3p | 0.99 | 0.77 | -4.22 | -3.59 | -0.63 | -1.55 | 6.2E-02 | 1.2E-01 |
| let-7i-5p | 0.42 | 0.55 | -0.16 | -0.51 | 0.35 | 1.27 | 6.8E-02 | 1.3E-01 |
| 15b-5p | 0.56 | 0.60 | -1.40 | -1.79 | 0.39 | 1.31 | 7.0E-02 | 1.3E-01 |
| 502-3p | 0.73 | 0.84 | -3.88 | -4.44 | 0.56 | 1.47 | 7.3E-02 | 1.3E-01 |
| 136-5p | 1.69 | 1.28 | -5.42 | -4.21 | -1.20 | -2.30 | 7.6E-02 | 1.4E-01 |
| 185-5p | 0.54 | 0.74 | 2.11 | 1.67 | 0.44 | 1.36 | 8.5E-02 | 1.5E-01 |
| 148b-3p | 0.42 | 0.39 | -0.62 | -0.87 | 0.25 | 1.19 | 9.2E-02 | 1.6E-01 |
| 339-5p | 1.24 | 1.30 | -4.23 | -3.46 | -0.77 | -1.71 | 1.0E-01 | 1.8E-01 |
| 195-5p | 1.43 | 1.19 | -5.24 | -6.04 | 0.80 | 1.74 | 1.1E-01 | 2.0E-01 |
| 92a-3p | 0.36 | 0.69 | 3.53 | 3.18 | 0.35 | 1.27 | 1.2E-01 | 2.0E-01 |
| 10b-5p | 0.71 | 0.98 | -2.22 | -1.73 | -0.49 | -1.40 | 1.4E-01 | 2.5E-01 |
| let-7b-5p | 0.48 | 0.65 | 0.52 | 0.21 | 0.31 | 1.24 | 1.6E-01 | 2.7E-01 |
| 142-5p | 0.78 | 0.62 | -1.81 | -1.47 | -0.34 | -1.26 | 1.7E-01 | 2.8E-01 |
| 150-5p | 1.21 | 0.91 | 0.13 | -0.37 | 0.50 | 1.42 | 1.7E-01 | 2.8E-01 |
| 146a-5p | 0.80 | 0.47 | -1.48 | -1.19 | -0.29 | -1.23 | 1.7E-01 | 2.9E-01 |
| 128-3p | 0.86 | 0.44 | -3.41 | -3.12 | -0.30 | -1.23 | 1.8E-01 | 2.9E-01 |
| 93-3p | 0.79 | 0.77 | -4.16 | -4.63 | 0.46 | 1.38 | 2.0E-01 | 3.2E-01 |
| 532-5p | 0.63 | 0.96 | -3.34 | -3.75 | 0.41 | 1.33 | 2.0E-01 | 3.2E-01 |
| 181a-5p | 0.85 | 1.14 | -2.36 | -1.87 | -0.49 | -1.40 | 2.1E-01 | 3.3E-01 |
| 425-3p | 1.06 | 0.90 | -4.61 | -4.16 | -0.45 | -1.37 | 2.3E-01 | 3.7E-01 |
| 194-5p | 0.54 | 0.94 | -3.04 | -3.38 | 0.34 | 1.27 | 2.6E-01 | 4.0E-01 |
| 127-3p | 2.42 | 1.15 | -6.54 | -5.22 | -1.33 | -2.51 | 2.6E-01 | 4.0E-01 |
| 543 | 1.04 | 1.12 | -5.75 | -4.83 | -0.92 | -1.89 | 2.7E-01 | 4.2E-01 |
| 497-5p | 0.88 | 0.98 | -3.82 | -3.45 | -0.37 | -1.29 | 2.8E-01 | 4.2E-01 |
| 22-5p | 0.78 | 0.61 | -3.28 | -3.55 | 0.27 | 1.20 | 2.8E-01 | 4.2E-01 |
| 7-1-3p | 1.35 | 1.48 | -5.73 | -4.95 | -0.78 | -1.72 | 2.9E-01 | 4.3E-01 |
| 320a | 0.34 | 0.94 | 2.19 | 2.49 | -0.31 | -1.24 | 2.9E-01 | 4.3E-01 |
| 24-3p | 0.41 | 0.47 | 1.28 | 1.45 | -0.17 | -1.13 | 2.9E-01 | 4.3E-01 |
| 18b-5p | 1.02 | 0.77 | -2.92 | -2.60 | -0.32 | -1.24 | 3.1E-01 | 4.5E-01 |
| 136-3p | 1.05 | 1.61 | -6.12 | -5.39 | -0.73 | -1.66 | 3.1E-01 | 4.6E-01 |
| 342-3p | 1.45 | 1.00 | -1.93 | -2.34 | 0.41 | 1.33 | 3.3E-01 | 4.7E-01 |
| 505-3p | 0.72 | 1.08 | -4.47 | -4.11 | -0.36 | -1.29 | 3.3E-01 | 4.7E-01 |
| 186-5p | 0.79 | 1.27 | -4.71 | -5.12 | 0.40 | 1.32 | 3.3E-01 | 4.7E-01 |
| 200c-3p | 1.09 | 1.64 | -6.14 | -6.86 | 0.72 | 1.65 | 3.4E-01 | 4.7E-01 |
| 584-5p | 1.07 | 1.38 | -6.26 | -5.81 | -0.45 | -1.36 | 3.6E-01 | 5.0E-01 |
| 122-5p | 1.06 | 1.15 | -0.10 | 0.26 | -0.37 | -1.29 | 3.6E-01 | 5.0E-01 |
| 148a-3p | 0.45 | 0.33 | -0.56 | -0.44 | -0.12 | -1.09 | 3.7E-01 | 5.1E-01 |
| 223-5p | 0.80 | 1.24 | -4.88 | -5.25 | 0.38 | 1.30 | 3.8E-01 | 5.1E-01 |
| 375 | 0.92 | 1.27 | -3.61 | -3.98 | 0.37 | 1.29 | 3.8E-01 | 5.1E-01 |
| 874-3p | 0.83 | 0.79 | -4.19 | -3.94 | -0.25 | -1.19 | 3.8E-01 | 5.1E-01 |
| 320b | 0.35 | 1.12 | -0.03 | 0.26 | -0.29 | -1.22 | 4.0E-01 | 5.3E-01 |
| 20b-5p | 0.95 | 1.52 | -6.08 | -6.62 | 0.54 | 1.45 | 4.1E-01 | 5.4E-01 |
| 365a-3p | 1.07 | 1.63 | -4.56 | -5.00 | 0.44 | 1.36 | 4.3E-01 | 5.6E-01 |
| 193a-5p | 0.85 | 1.26 | -5.54 | -5.88 | 0.34 | 1.26 | 4.3E-01 | 5.6E-01 |
| 99b-5p | 1.14 | 1.68 | -4.75 | -4.34 | -0.41 | -1.33 | 4.7E-01 | 6.1E-01 |
| 320c | 0.66 | 1.24 | -1.17 | -0.89 | -0.28 | -1.21 | 4.8E-01 | 6.2E-01 |
| 382-5p | 1.03 | 1.21 | -5.30 | -4.95 | -0.35 | -1.27 | 5.1E-01 | 6.4E-01 |
| 339-3p | 0.77 | 0.89 | -4.83 | -4.63 | -0.20 | -1.15 | 5.5E-01 | 6.9E-01 |
| 205-5p | 1.26 | 1.43 | -5.35 | -5.05 | -0.30 | -1.23 | 5.6E-01 | 7.0E-01 |
| 34a-5p | 0.97 | 1.42 | -3.08 | -3.37 | 0.29 | 1.22 | 5.7E-01 | 7.1E-01 |
| 28-3p | 0.91 | 1.33 | -5.10 | -4.85 | -0.25 | -1.19 | 5.8E-01 | 7.2E-01 |
| 130b-3p | 0.53 | 1.34 | -4.89 | -5.11 | 0.22 | 1.17 | 5.9E-01 | 7.2E-01 |
| 1260a | 0.94 | 1.11 | -3.21 | -2.98 | -0.23 | -1.17 | 6.0E-01 | 7.2E-01 |
| 629-5p | 1.01 | 1.30 | -3.70 | -3.93 | 0.23 | 1.17 | 6.0E-01 | 7.2E-01 |
| 30a-5p | 0.98 | 0.82 | -3.50 | -3.66 | 0.16 | 1.12 | 6.1E-01 | 7.3E-01 |
| 877-5p | 1.06 | 1.15 | -5.30 | -5.00 | -0.29 | -1.22 | 6.3E-01 | 7.4E-01 |
| 30e-3p | 1.23 | 1.26 | -5.89 | -5.65 | -0.24 | -1.18 | 6.3E-01 | 7.4E-01 |
| 200a-3p | 1.30 | 1.85 | -5.77 | -5.25 | -0.52 | -1.43 | 6.3E-01 | 7.4E-01 |
| 501-3p | 1.12 | 1.07 | -5.40 | -5.17 | -0.23 | -1.17 | 6.4E-01 | 7.4E-01 |
| let-7g-5p | 0.49 | 0.42 | 0.34 | 0.41 | -0.07 | -1.05 | 6.4E-01 | 7.4E-01 |
| 324-5p | 0.85 | 1.72 | -4.50 | -4.78 | 0.28 | 1.21 | 6.6E-01 | 7.6E-01 |
| 361-5p | 0.59 | 1.10 | -2.09 | -2.21 | 0.13 | 1.09 | 7.1E-01 | 8.1E-01 |
| 424-5p | 0.55 | 0.60 | -0.87 | -0.80 | -0.07 | -1.05 | 7.2E-01 | 8.2E-01 |
| 144-5p | 1.10 | 0.85 | -1.96 | -2.07 | 0.11 | 1.08 | 7.3E-01 | 8.2E-01 |
| 30d-5p | 0.20 | 0.44 | -0.50 | -0.46 | -0.05 | -1.03 | 7.4E-01 | 8.2E-01 |
| 483-5p | 1.39 | 2.42 | -4.78 | -4.54 | -0.24 | -1.18 | 7.5E-01 | 8.4E-01 |
| 423-5p | 0.34 | 0.58 | 0.33 | 0.29 | 0.05 | 1.03 | 7.9E-01 | 8.8E-01 |
| let-7b-3p | 1.02 | 1.13 | -5.06 | -5.16 | 0.10 | 1.07 | 8.1E-01 | 8.8E-01 |
| 17-5p | 1.03 | 1.02 | -4.83 | -4.73 | -0.09 | -1.07 | 8.1E-01 | 8.8E-01 |
| 885-5p | 1.07 | 1.08 | -5.08 | -5.17 | 0.10 | 1.07 | 8.1E-01 | 8.8E-01 |
| 362-3p | 0.89 | 0.79 | -3.92 | -3.98 | 0.06 | 1.04 | 8.4E-01 | 9.0E-01 |
| 454-3p | 1.40 | 1.18 | -5.05 | -5.14 | 0.09 | 1.06 | 8.5E-01 | 9.0E-01 |
| 154-5p | 1.01 | 1.54 | -5.00 | -4.88 | -0.12 | -1.09 | 8.5E-01 | 9.0E-01 |
| 133a-3p | 1.11 | 1.46 | -6.06 | -5.95 | -0.11 | -1.08 | 8.5E-01 | 9.0E-01 |
| 106b-3p | 1.74 | 1.49 | -6.48 | -6.33 | -0.15 | -1.11 | 8.5E-01 | 9.0E-01 |
| 574-3p | 0.93 | 1.29 | -4.54 | -4.63 | 0.09 | 1.06 | 8.6E-01 | 9.0E-01 |
| 92b-3p | 1.03 | 0.71 | -6.86 | -6.92 | 0.06 | 1.05 | 8.9E-01 | 9.3E-01 |
| 484 | 0.44 | 0.90 | -1.64 | -1.61 | -0.03 | -1.02 | 9.1E-01 | 9.4E-01 |
| 132-3p | 1.05 | 0.80 | -4.24 | -4.28 | 0.04 | 1.03 | 9.2E-01 | 9.4E-01 |
| 141-3p | 0.86 | 1.47 | -5.96 | -5.90 | -0.06 | -1.04 | 9.3E-01 | 9.5E-01 |
| 320d | 0.55 | 0.51 | -1.82 | -1.79 | -0.02 | -1.02 | 9.3E-01 | 9.5E-01 |
| 146b-5p | 1.45 | 1.28 | -5.84 | -5.87 | 0.03 | 1.02 | 9.6E-01 | 9.7E-01 |
| 155-5p | 0.84 | 1.05 | -4.72 | -4.71 | 0.00 | -1.00 | 9.9E-01 | 1.0E+00 |
| 133b | 0.87 | 1.54 | -4.74 | -4.74 | 0.00 | 1.00 | 1.0E+00 | 1.0E+00 |
| 208a-3p | ND | ND | -7.75 | ND | ND | ND | ND | ND |

Note: BH adj: Benjamini-Hochberg adjusted *P*-value; dCq: Normalized quantitation cycles; ddCq: relative quantification between the groups; ND: Not determined; SD: Standard deviation
